# Characterization of Monoclonal Antibody LpMab-3 Recognizing Sialylated Glycopeptide of Podoplanin

**DOI:** 10.1089/mab.2014.0087

**Published:** 2015-02-01

**Authors:** Hiroharu Oki, Satoshi Ogasawara, Mika Kato Kaneko, Michiaki Takagi, Masanori Yamauchi, Yukinari Kato

**Affiliations:** ^1^ Department of Regional Innovation, Tohoku University Graduate School of Medicine, Sendai, Miyagi, Japan.; ^2^ Department of Orthopaedic Surgery, Yamagata University Faculty of Medicine, Yamagata, Japan.; ^3^ Department of Anesthesiology and Perioperative Medicine, Tohoku University Graduate School of Medicine, Sendai, Miyagi, Japan.

## Abstract

Podoplanin (PDPN/Aggrus/T1α/gp36/OTS-8), a type I transmembrane sialoglycoprotein, is involved in platelet aggregation, cell invasion, and cancer metastasis. Podoplanin expression in cancer cells or cancer-associated fibroblasts was reported to be involved in poor prognosis of several cancers. Furthermore, podoplanin is expressed in lymphatic endothelial cells or lung type I alveolar cells. Although many anti-podoplanin monoclonal antibodies (MAbs), such as NZ-1 and D2–40, have been established, almost all anti-podoplanin MAbs are produced against a platelet aggregation-inducing (PLAG) domain. In this study, we produced and characterized a novel anti-podoplanin monoclonal antibody, LpMab-3, the epitope of which is a sialylated glycopeptide of podoplanin. We identified the minimum epitope of LpMab-3 as Thr76–Glu81 of human podoplanin, which is different from PLAG domain, using Western blot analysis and flow cytometry. Immunohistochemical analysis showed that LpMab-3 is useful for detecting lung type I alveolar cells and lymphatic endothelial cells. Because LpMab-3 detects only sialylated podoplanin, it could be useful for uncovering the physiological function of sialylated human podoplanin.

## Introduction

Podoplanin (PDPN/Aggrus/T1α/gp36/OTS-8) is a platelet aggregation-inducing mucin-type glycoprotein that is involved in cancer metastasis.^(1,2)^ Expression of podoplanin has been reported in many cancers including malignant gliomas, lung cancer, esophageal cancer, malignant mesotheliomas, testicular tumors, bladder cancer, and osteosarcoma.^(1,3–14)^ Moreover, podoplanin expression in cancer-associated fibroblasts (CAFs) was reported to be involved in poor prognosis of several cancers.^(15–20)^ We previously identified C-type lectin-like receptor-2 (CLEC-2) as an endogenous receptor of podoplanin^(21,22)^ and recently performed comparative crystallographic studies of podoplanin in complex with CLEC-2.^(23)^ The interaction with CLEC-2 was mainly observed at Glu47 and Asp48 in the PLAG3 domain and the α2–6 linked sialic acid at Thr52 of podoplanin.

Anti-podoplanin MAbs with high sensitivity and specificity are necessary to analyze the physiological function of podoplanin in normal tissues and cancers. Although many anti-podoplanin MAbs have been produced, almost all anti-podoplanin MAbs react with a platelet aggregation-inducing (PLAG) domain of human podoplanin.^(7,24–28)^ Rabbit polyclonal antibodies produced by immunizing recombinant rat podoplanin also recognize PLAG domains, which were shown to be immunodominant antigenic sites.^(29)^ We recently established the platform to produce cancer-specific MAbs (CasMabs).^(30)^ In this study, we produced and characterized a novel anti-podoplanin monoclonal antibody, LpMab-3, one of non-CasMabs.

## Materials and Methods

### Cell lines and tissues

Chinese hamster ovary (CHO)-K1, glycan-deficient CHO cell lines (Lec1, Lec2, and Lec8), LN229, NCI-H226, and P3U1 were purchased from the American Type Culture Collection (ATCC, Manassas, VA). Human lymphatic endothelial cells (LEC) were obtained from Cambrex (Walkersville, MD). The human glioblastoma cell line LN319 was donated by Dr. Webster K. Cavenee (Ludwig Institute for Cancer Research, San Diego, CA). CHO-K1, Lec1, Lec2, Lec8, and LN229 were transfected with human podoplanin plasmids (CHO/hPDPN, Lec1/hPDPN, Lec2/hPDPN, Lec8/hPDPN, and LN229/hPDPN) using Lipofectamine 2000 (Life Technologies, Carlsbad, CA) according to the manufacturer's instructions.^(30)^ CHO-K1, Lec1, Lec2, Lec8, NCI-H226, and P3U1 were cultured in RPMI 1640 medium (Wako Pure Chemical Industries, Osaka, Japan), and LN229 and LN319 were cultured in Dulbecco's Modified Eagle's Medium (DMEM) medium (Wako Pure Chemical Industries), supplemented with 10% heat-inactivated fetal bovine serum (FBS; Life Technologies), 2 mM L-glutamine (Life Technologies), 100 U/mL of penicillin, and 100 μg/mL of streptomycin (Life Technologies) at 37°C in a humidified atmosphere of 5% CO_2_ and 95% air. L-proline (0.04 mg/mL) was added for Lec1, Lec2, and Lec8. LEC was cultured in endothelial cell medium EGM-2MV supplemented with 5% FBS (Cambrex). Tissue microarrays were purchased from Cybrdi (Frederick, MD).

### Antibodies

LpMab-7 (mouse IgG_1_, kappa), NZ-1 (rat IgG_2a_, lambda), r2336 (rabbit polyclonal), and RMab-3 (mouse IgG_1_, kappa) were developed previously in our laboratories.^(7,24,30,31)^ Anti-FLAG tag MAb (1E6) and anti-β-actin MAb (AC15) were purchased from Wako Pure Chemical Industries and Sigma-Aldrich (St. Louis, MO), respectively.

### Hybridoma production

BALB/c mice were immunized by intraperitoneal (i.p.) injection of 1×10^8^ LN229/hPDPN cells together with Imject Alum (Thermo Fisher Scientific, Waltham, MA). After several additional immunizations, a booster injection was given i.p. 2 days before spleen cells were harvested. The spleen cells were fused with P3U1 cells using GenomONE-CF (Ishihara Sangyo Kaisha, Osaka, Japan). The hybridomas were grown in RPMI medium with hypoxanthine, aminopterin, and thymidine selection medium supplement (Life Technologies). The culture supernatants were screened using enzyme-linked immunosorbent assay (ELISA) for binding to recombinant human podoplanin purified from LN229/hPDPN cells. Next, flow cytometry was performed against LN229/hPDPN and LN229 cells.

### Enzyme-linked immunosorbent assay

Purified proteins were immobilized on Nunc Maxisorp 96-well immunoplates (Thermo Fisher Scientific) at 1 μg/mL for 30 min.^(30)^ After blocking with SuperBlock T20 (PBS) blocking buffer (Thermo Fisher Scientific), the plates were incubated with culture supernatant or purified MAbs (1 μg/mL) followed by 1:1000 diluted peroxidase-conjugated anti-mouse IgG (Dako, Glostrup, Denmark). The enzymatic reaction was conducted with a 1-Step Ultra TMB-ELISA (Thermo Fisher Scientific). The optical density was measured at 655 nm using an iMark microplate reader (Bio-Rad Laboratories, Philadelphia, PA). These reactions were performed with a volume of 50 μL at 37°C.

### Production of podoplanin mutants

The amplified human podoplanin cDNA was subcloned into a pcDNA3 vector (Life Technologies) and a FLAG epitope tag was added at the C-terminus. Substitution of amino acids to alanine in podoplanin was performed using a QuikChange Lightning site-directed mutagenesis kit (Agilent Technologies, Santa Clara, CA).^(30,32)^ CHO-K1 cells were transfected with the plasmids using a Gene Pulser Xcell electroporation system (Bio-Rad Laboratories).

### Flow cytometry

Cell lines were harvested by brief exposure to 0.25% Trypsin/1 mM EDTA (Wako Pure Chemical Industries).^(22)^ After washing with phosphate-buffered saline (PBS), the cells were treated with primary antibodies (1 μg/mL) for 30 min at 4°C, followed by treatment with Oregon green-conjugated anti-mouse IgG (Life Technologies), Alexa Fluor 488 conjugated anti-mouse IgG (Cell Signaling Technology, Danvers, MA), or Alexa Fluor 488 conjugated anti-rat IgG (Cell Signaling Technology). Fluorescence data were collected using a FACS Calibur flow cytometer (BD Biosciences, Braintree, MA) or a Cell Analyzer EC800 (Sony, Tokyo, Japan).

### Western blot analyses

Cell lysates (10 μg) were boiled in SDS sample buffer (Nacalai Tesque, Kyoto, Japan).^(33)^ The proteins were electrophoresed on 5–20% polyacrylamide gels (Wako Pure Chemical Industries) and were transferred onto a PVDF membrane (EMD Millipore, Billerica, MA). After blocking with SuperBlock T20 (PBS) Blocking Buffer, the membrane was incubated with primary antibodies (1 μg/mL), and then with peroxidase-conjugated secondary antibodies (Dako, Glostrup, Denmark; 1:1000 diluted), and developed with the ECL-plus reagent (Thermo Fisher Scientific) using a Sayaca-Imager (DRC, Tokyo, Japan).

### Immunohistochemical analyses

Four-μm-thick histologic sections were deparaffinized in xylene and rehydrated. Then they were autoclaved in citrate buffer (pH 6.0; Dako) for 20 min. Sections were incubated with 5 μg/mL of LpMab-3 overnight at 4°C followed by treatment with an Envision+ kit (Dako). Color was developed using 3,3-diaminobenzidine tetrahydrochloride (DAB; Dako) for 10 min, and the sections were counterstained with hematoxylin (Wako Pure Chemical Industries).

### Affinity determination by surface plasmon resonance

To determine the affinity, recombinant podoplanin-Fc was immobilized on the surface of chips for analysis using the BIAcore 3000 system (GE Healthcare, Piscataway, NJ). The running buffer was 10 mM HEPES, 150 mM NaCl, and 0.005% v/v Surfactant P20 (BR-1003–68, pH 7.4; GE Healthcare). LpMab-3 was passed over the biosensor chip, and the affinity rate constants (association rate constant, *k*_assoc_, and disassociation rate constant, *k*_diss_) were determined by nonlinear curve-fitting using the Langmuir one-site binding model of the BIAevaluation software (GE Healthcare). The affinity constant (*K*_A_) at equilibrium was calculated as *K*_A_=*k*_assoc_/*k*_diss_, and the dissociate constant (*K*_D_) was determined as 1/*K*_A_.

## Results

### Production and characterization of novel anti-podoplanin monoclonal antibody LpMab-3

**FIG. 1.**
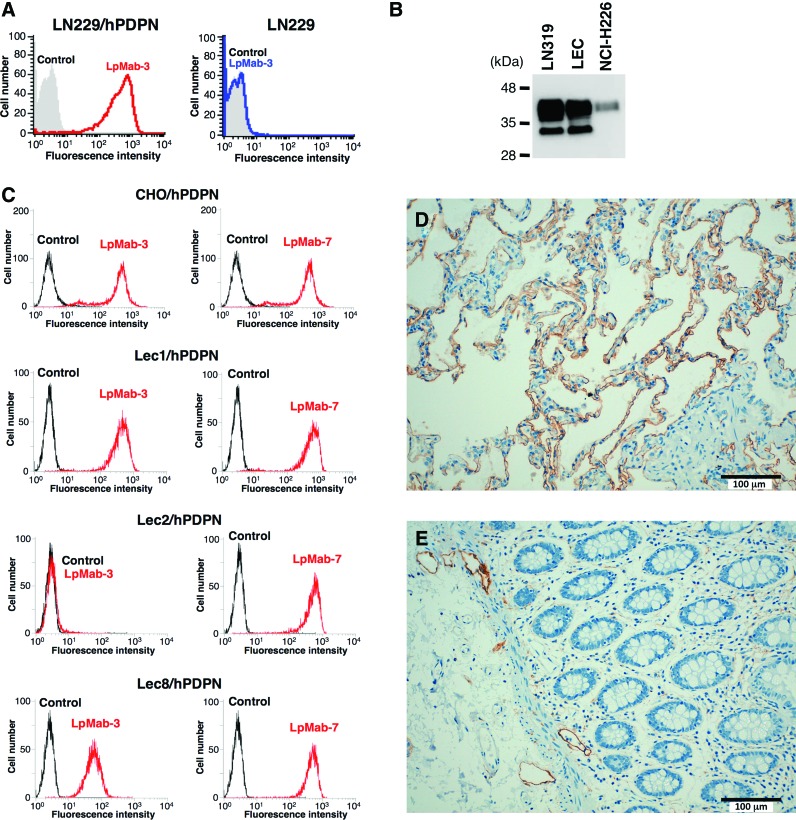
(**A**) Flow cytometric analysis by LpMab-3 against LN229/hPDPN and LN229. Cell lines were treated with LpMab-3 (1 μg/mL) for 30 min at 4°C, followed by treatment with Oregon green-conjugated anti-mouse IgG. Fluorescence data were collected using a FACS Calibur flow cytometer. (**B**) Western blot analysis by LpMab-3. Total cell lysate were electrophoresed on 5–20% polyacrylamide gels and transferred onto a PVDF membrane. After blocking, the membrane was incubated with 1 μg/mL of LpMab-3 and then with peroxidase-conjugated anti-mouse IgG; the membrane was detected using a Sayaca-Imager. (**C**) Flow cytometric analysis by LpMab-3 and LpMab-7 against glycan-deficient podoplanin-expressing CHO cell lines. Cell lines were treated with LpMab-3 and LpMab-7 (1 μg/mL) for 30 min at 4°C, followed by treatment with Alexa Fluor 488 conjugated anti-mouse IgG. Fluorescence data were collected using a Cell Analyzer EC800. (**D, E**) Immunohistochemical analysis against normal tissues using LpMab-3. Sections of normal lung (**D**) and normal colon (**E**) were incubated with 5 μg/mL of LpMab-3, followed by Envision+ kit. Color was developed using DAB and counterstained with hematoxylin.

To develop novel anti-podoplanin MAbs, we immunized mice with LN229/hPDPN cells. The culture supernatants were screened using ELISA for binding to recombinant human podoplanin purified from LN229/hPDPN cells. After limiting the dilution of the hybridomas, LpMab-3 (IgG_1_, kappa) was established. LpMab-3 reacted with LN229/hPDPN, not with LN229, a podoplanin-negative cell line (Fig. 1A). Furthermore, LpMab-3 detected endogenous podoplanin, which is expressed in LN319 (a glioblastoma cell line), a lymphatic endothelial cell (LEC), and NCI-H226 (a malignant mesothelioma cell line) (Fig. 1B). We next performed flow cytometric analyses using LpMab-3 against several glycan-deficient podoplanin transfectants (Fig. 1C). LpMab-7, which was used as a positive control, reacted with all podoplanin transfectants. In contrast, LpMab-3 did not react with Lec2/hPDPN (sialic acid-deficient), although it reacted with CHO/hPDPN, Lec1/hPDPN (*N*-glycan deficient), or Lec8/hPDPN (*O*-glycan deficient) cells, indicating that LpMab-3 recognizes sialylated podoplanin.

We next performed a kinetic analysis of the interaction of LpMab-3 with a recombinant podoplanin using surface plasmon resonance (BIAcore). Determination of the association and dissociation rates from the sensorgrams revealed that *k*_assoc_ of 1.12×10^4^ (mol/L-s)^−1^ and *k*_diss_ of 9.49×10–^4^ s^−1^. The *K*_A_ at binding equilibrium, calculated as *K*_A_=*k*_assoc_/*k*_diss_, was 1.18×10^7^ (mol/L)^−1^, *K*_D_=1/*K*_A_=8.5×10–^8^ M. The affinity of LpMab-3 calculated by BIAcore is about 200 times lower than that of NZ-1 (*K*_D_: 4.0×10–^10^ M).^(34)^

### Immunohistochemical analysis against podoplanin-expressing normal tissues using LpMab-3

We investigated the podoplanin expression in normal lung and colon. As shown in Figure 1D, LpMab-3 detected type I alveolar cells. In our previous study, NZ-1 could not detect type I alveolar cells in immunohistochemistry^(10)^; therefore, LpMab-3 is more useful for detecting type I alveolar cells compared with previous anti-podoplanin MAbs. LpMab-3 also detects lymphatic endothelial cells of normal colon (Fig. 1E). Taken together, LpMab-3 is useful for immunohistochemistry using paraffin-embedded tissues.

### Epitope mapping by Western blot analysis and flow cytometry

**FIG. 2.**
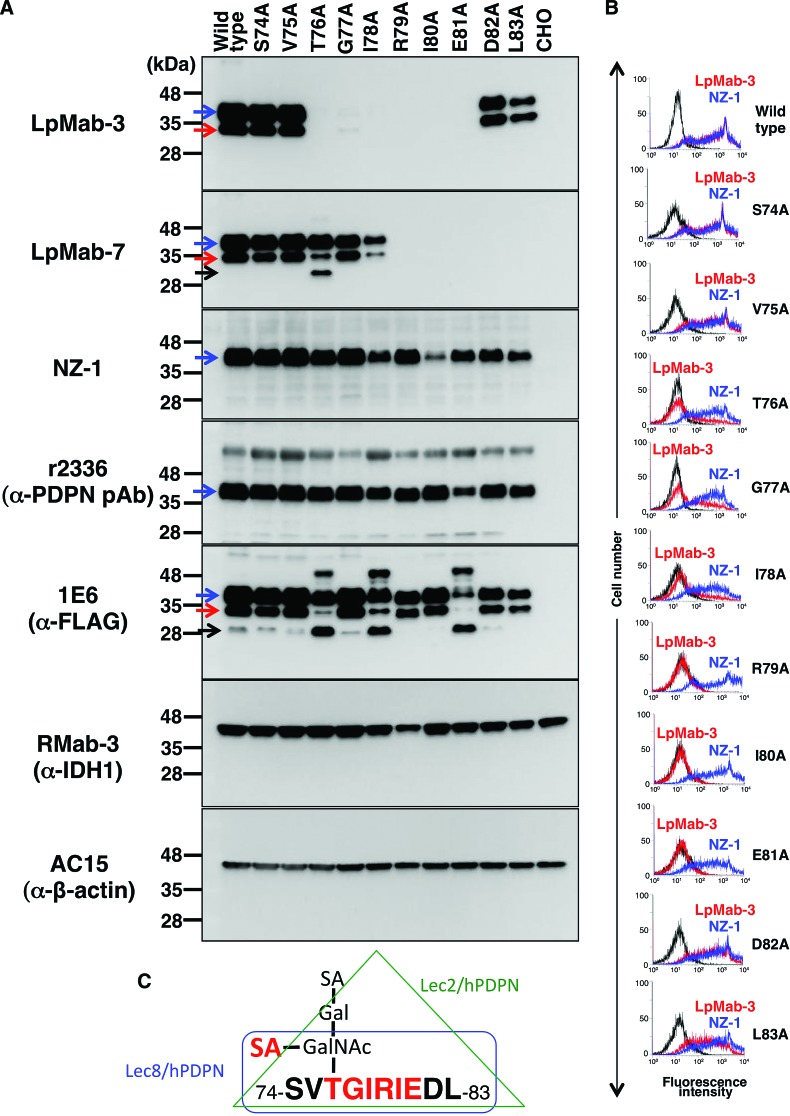
Epitope mapping of LpMab-7 by Western blot analysis and flow cytometry. (**A**) Western blotting by LpMab-3, LpMab-7, NZ-1, r2336, 1E6, RMab-3 (α-IDH1), and AC-15 (α-β-actin). Total cell lysate were electrophoresed on 5–20% polyacrylamide gels and transferred onto a PVDF membrane. After blocking, the membrane was incubated with 1 μg/mL of primary antibodies and then with peroxidase-conjugated secondary antibodies; the membrane was detected using a Sayaca-Imager. Blue arrow, 40 kDa band (glycosylated); red arrow, 30 kDa band (glycosylated); black arrow, 25 kDa band (non-glycosylated). (**B**) Point mutants of human podoplanin were treated with NZ-1 and LpMab-3 (1 μg/mL) for 30 min at 4°C, followed by treatment with Alexa Fluor 488 conjugated anti-rat IgG and anti-mouse IgG, respectively. Fluorescence data were collected using a Cell Analyzer EC800. (**C**) *TGIRIE* sequence and α2–6 linked sialic acid are the critical epitope of LpMab-3.

To determine the LpMab-3 epitope, we first performed Western blot analysis. LpMab-3 reaction was lost in point mutations of 76–81 amino acids (Fig. 2A). This epitope includes Thr76, the only Ser/Thr residue, indicating that Thr76 is sialylated and is essential for LpMab-3 recognition. In contrast, LpMab-7 reaction was lost in point mutations of 79–83 amino acids. Interestingly, 79–81 amino acids are included as epitopes of both LpMab-3 and LpMab-7. Furthermore, both LpMab-3 and LpMab-7 detects two bands (40 kDa and 30 kDa; glycosylated podoplanin), whereas NZ-1 (a rat anti-PLAG domain MAb) and r2336 (a rabbit anti-N-terminus of podoplanin polyclonal antibody [pAb]) detect only one band (40 kDa). Anti-FLAG tag MAb detects both two bands of glycosylated podoplanin; therefore, both LpMab-3 and LpMab-7 are more sensitive against podoplanin compared with anti-N-terminus antibodies (NZ-1 and r2336). LpMab-7 and anti-FLAG tag MAbs also detected non-glycosylated podoplanin (25 kDa) of several podoplanin point mutants.

We next performed flow cytometric analysis using LpMab-3 and NZ-1 MAbs against the same point mutants of podoplanin. The results revealed that LpMab-3 did not react with R79A, I80A, and E81A, and weakly reacted with T76A, G77A, and I78A (Fig. 2B), indicating that *TGIRIE* sequence is the minimum epitope, and Arg79, Ile80, and Glu81 are much more critical residues for LpMab-3 epitopes (Fig. 2C).

## Discussion

Podoplanin is expressed in normal tissues such as lymphatic endothelial cells, lung type I alveolar cells, epidermal keratinocytes, kidney podocytes, and fibroblastic reticular cells (FRCs) of lymph nodes.^(35,36)^ Recently, several physiological functions of podoplanin have been reported. The activation of CLEC-2 by podoplanin (the signal from podoplanin to CLEC-2) rearranges the actin cytoskeleton in dendritic cells to promote efficient motility along stromal surfaces.^(37)^ In contrast, the signal from CLEC-2 to podoplanin controls the contractility of FRCs and lymph node microarchitecture.^(38)^ The physical elasticity of lymph nodes is maintained by podoplanin of stromal FRCs and its modulation by CLEC-2 of dendritic cells.^(39)^ Although we have shown that podoplanin possesses platelet-aggregating activity via CLEC-2 in cancer models, podoplanin-CLEC-2 interaction is also important for embryonic blood-lymphatic vascular separation using platelet aggregation.^(1,2,21,22,40–42)^ The local sphingosine-1-phosphate release after podoplanin-CLEC-2-mediated platelet activation is critical for the integrity of high endothelial venules during immune responses.^(43)^ Furthermore, the development of ectopic lymphoid follicles is dependent on Th17-expressing podoplanin.^(44)^ Taken together, the reciprocal interaction between podoplanin and CLEC-2 is important in many physiological functions. Therefore, development of novel anti-podoplanin MAbs, the epitopes of which are different, is still important.

LpMab-3 possesses a unique epitope that is completely different from that previously reported for anti-podoplanin MAbs such as NZ-1 and D2–40. The epitope is similar to that of LpMab-7; however, LpMab-3 needs sialylation of Thr76. Because only α2–6 linked sialic acid was attached to podoplanin on Lec8/hPDPN,^(40)^ LpMab-3 epitope may include α2–6 linked sialic acid, not α2–3 linked sialic acid. Therefore, LpMab-3 is useful for distinguishing Thr76-sialylated from Thr76-nonsialylated podoplanin. However, the binding affinity of LpMab-3 was shown to be lower than that of NZ-1. Because the binding affinity of antibodies is critical for antibody-based cancer therapy, affinity maturation of LpMab-3 should be considered in the future. Using the CasMab method, we can obtain not only cancer-specific MAbs (CasMabs) but also non-CasMabs such LpMab-3 and LpMab-7. Of interest, non-CasMabs, such as LpMab-3, also include the glycan within those epitopes.^(30)^ Although antibody-dependent cellular cytotoxicity (ADCC) and complement-dependent cytotoxicity (CDC) activities are very important for an antibody-based molecular targeting therapy, we could not investigate these activities because the subclass of LpMab-3 is mouse IgG_1_. The conversion of subclass into human IgG_1_ or mouse IgG_2a_ is necessary to demonstrate ADCC/CDC activities.
